# An estimate of fitness reduction from mutation accumulation in a mammal allows assessment of the consequences of relaxed selection

**DOI:** 10.1371/journal.pbio.3002795

**Published:** 2024-09-26

**Authors:** Jobran Chebib, Anika Jonas, Eugenio López-Cortegano, Sven Künzel, Diethard Tautz, Peter D. Keightley

**Affiliations:** 1 Institute of Ecology and Evolution, University of Edinburgh, Edinburgh, United Kingdom; 2 Department for Evolutionary Genetics, Max Planck Institute for Evolutionary Biology, Plön, Germany; University of Bath, UNITED KINGDOM OF GREAT BRITAIN AND NORTHERN IRELAND

## Abstract

Each generation, spontaneous mutations introduce heritable changes that tend to reduce fitness in populations of highly adapted living organisms. This erosion of fitness is countered by natural selection, which keeps deleterious mutations at low frequencies and ultimately removes most of them from the population. The classical way of studying the impact of spontaneous mutations is via mutation accumulation (MA) experiments, where lines of small effective population size are bred for many generations in conditions where natural selection is largely removed. Such experiments in microbes, invertebrates, and plants have generally demonstrated that fitness decays as a result of MA. However, the phenotypic consequences of MA in vertebrates are largely unknown, because no replicated MA experiment has previously been carried out. This gap in our knowledge is relevant for human populations, where societal changes have reduced the strength of natural selection, potentially allowing deleterious mutations to accumulate. Here, we study the impact of spontaneous MA on the mean and genetic variation for quantitative and fitness-related traits in the house mouse using the MA experimental design, with a cryopreserved control to account for environmental influences. We show that variation for morphological and life history traits accumulates at a sufficiently high rate to maintain genetic variation and selection response. Weight and tail length measures decrease significantly between 0.04% and 0.3% per generation with narrow confidence intervals. Fitness proxy measures (litter size and surviving offspring) decrease on average by about 0.2% per generation, but with confidence intervals overlapping zero. When extrapolated to humans, our results imply that the rate of fitness loss should not be of concern in the foreseeable future.

## Abstract 2

Spontaneous mutations tend to reduce fitness in populations of living organisms, but this erosion of fitness is countered by natural selection. This study uses the first mutation accumulation experiment in a mammal to show that even in the absence of natural selection, the rate of fitness loss should not be of concern, which is reassuring for humans.

## Introduction

Variation from spontaneous mutation forms the origin of all genetic variation. As such, new mutations have a central role in evolutionary genetics by fueling selection response and evolutionary adaptation. In addition, the variation arising from mutation has been implicated in a wide range of evolutionary phenomena, including, for example, the evolution of sex and recombination [1].

The rate of new spontaneous mutations arising in the genome can be quantified by comparing the sequences of related individuals. Advances in DNA sequencing technology have thereby enabled estimates of rates of mutation to be made across a wide range of taxonomic groups [2–5]. In humans, for example, comparison of the genome sequences of trios of parents and their offspring has shown that the mutation rate per nucleotide site per generation is about 10^−8^, implying that each newborn carries an average of about 70 new mutations in the entire diploid genome [6].

The impact of spontaneous mutations on quantitative variation, including variation for fitness and its components, has been more difficult to quantify. This is principally because new variation arising from spontaneous mutations has been shown to be a tiny proportion of the environmental variation and therefore has a negligible impact on the heritability in each generation [7]. Also, in the case of outbred individuals, spontaneous mutational variation is also a tiny proportion of the existing (standing) genetic variation for quantitative traits and these two sources of variation are hard to distinguish from one another.

Experimental approaches to measure quantitative variation arising from new spontaneous mutations therefore invariably involve the accumulation of mutations over many generations. Mutations are typically accumulated in lines or chromosomes descended from progenitor individuals, accompanied by the measurement of changes of phenotypic and molecular variation among replicated mutation accumulation (MA) lines or between selection lines. The progenitor individuals used to initiate the experimental lines are usually inbred in order to minimise standing genetic variation. Outbred individuals have also been used as the experimental progenitors [8,9], but this approach has been questioned on the grounds that any changes of the standing genetic variation that occur as the experiment progresses will tend to swamp new mutational variation [10]. This could be caused, for example, by natural selection or the breakdown of linkage disequilibrium.

The new genetic variation arising in one generation from mutation, *V*_*M*_, is usually expressed as a proportion of the existing phenotypic variation, *V*_*P*_, i.e., the mutational heritability = *h*^2^_*M*_ = *V*_*M*_/*V*_*P*_, or can also be expressed scaled by the trait mean (*M*) as the mutational coefficient of variation, *CV*_*M*_ = √*V*_*M*_/*M*, a measure of the “evolvability” of a trait [11]. In an inbred population, where the phenotypic variance and environmental variance (*V*_*E*_) should be nearly equivalent, the new heritability arising from mutation is often expressed as *h*^2^_*M*_ = *V*_*M*_/*V*_*E*_. Mutational heritability in plants and invertebrates is commonly of the order of 0.1% [12,13]. Although this is a tiny amount of variation, it is detectable after many generations of between-subline mutation accumulation, given sufficient replication. Such an input of variation can also lead to the maintenance of high heritabilities for quantitative traits under a neutral model, even in a moderately sized population, implying that natural selection acts to limit quantitative variation [7].

In vertebrates, however, there is little information on the magnitude of mutational variation. The only estimates are from mice, and are either based on the divergence for skeletal traits between pairs of inbred lines [14] or on the response in lines subject to divergent artificial selection on body size [15]. These experiments suggest that *h*^2^_*M*_ may be as high as approximately 1%. If applicable to commercial populations of farm animals, this amount of new variation would substantially impact long-term artificial selection response [16], albeit with the potential for undesirable pleiotropic side effects [17].

Changes in the mean values of quantitative traits, particularly for traits related to fitness have been at least as difficult to measure as changes in genetic variance. This has been at least in part due to the fact that a contemporary mutation-free control population has often not been available. The first experiments to measure changes in mean fitness as a consequence of mutation accumulation were in *Drosophila melanogaster* and employed balancer chromosomes to allow mutations to accumulate in the heterozygous state over many generations. These experiments implied that viability decays by at least 1% per generation as a consequence of spontaneous mutation accumulation. However such experiments have been challenged on the basis of the stability of the control chromosomes, against which viability change is measured [18,19]. Later MA experiments in *Caenorhabditis elegans* employed cryopreserved (presumably) mutation-free control populations, and resulted in much smaller losses of fitness per generation, of the order of 0.1% per generation [20,21]. Crucially, this loss of fitness essentially disappeared if mutation accumulation was allowed to occur in a population as small as 10 individuals implying that most mutational variation for fitness comprises of strongly deleterious mutations that are rapidly removed, even in small populations [21]. Other experiments in *Arabidopsis*, *Chlamydomonas*, and *Tetrahymena* either detected very small changes of fitness per generation, or inferred the presence of a large fraction of advantageous mutations in the environments in which the MA lines were at assayed [22–24].

There is essentially no information on rates of change of fitness traits from mutation accumulation in vertebrates. This is an important gap in our knowledge for two principal reasons. First, it is postulated that MA could be a threat to populations whose size has been reduced by human activities, where deleterious mutations can drift to fixation [25], or in captive populations of wild animals, where natural selection has been relaxed. Second, the new variation arising from mutation could substantially impact response to artificial selection [16] and could maintain heritability and selection response to changing environmental conditions in natural populations.

In humans, a potential role for new mutations reducing fitness over a measurable timescale has been postulated since early on in the history of genetics [26]. Humans have long generation times and the human mutation rate is higher than many other species [3,27]. The resulting genomic deleterious mutation rate has led to debate about long term persistence of human populations [28–31]. There has also been concern about the possibility of deleterious mutations accumulating in human populations over a shorter timescale, since recent improvements in living conditions and health care have undoubtedly reduced the strength of natural selection against deleterious mutations in some human populations [26,27,32]. A relaxation of selection will eventually lead to a new, higher equilibrium frequency for deleterious mutations. If selection was completely relaxed, the rate of genetic degradation per generation from mutation accumulation would be equal to the product of the genomic mutation rate per generation and the average fitness effect of a mutation in the heterozygous state. Under the scenario of a complete removal of natural selection, any loss would be realised when the environment deteriorates and natural selection is restored in the future. Based on information on rates of change of fitness in MA experiments in invertebrates, it has been argued that the rate of fitness erosion in humans could be as high as 5% and at least 1% per generation [33]. However, the phenotypic consequences of a relaxation of selection and a consequent accumulation of deleterious mutations have not been measured in any mammalian species.

**Fig 1:**
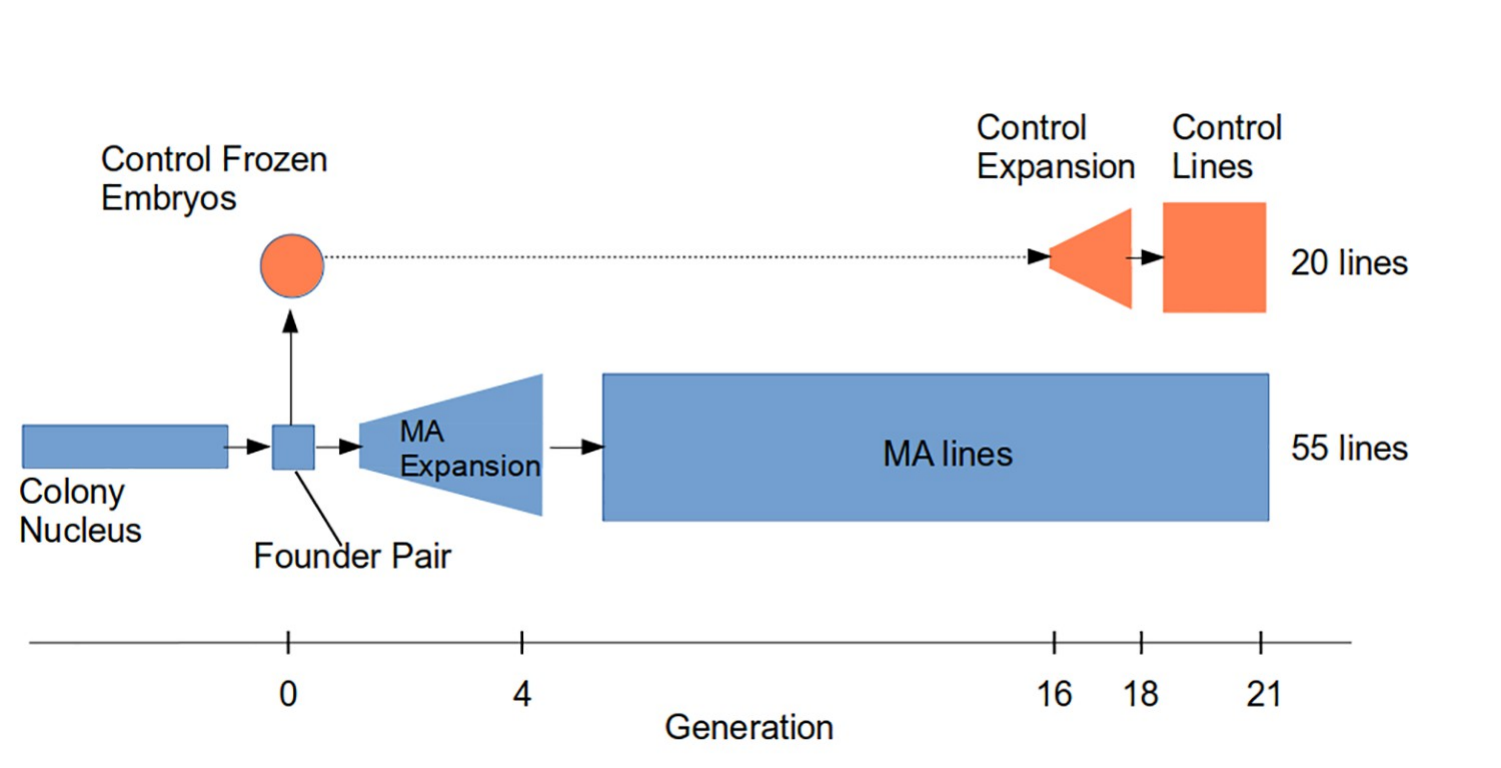
Overall design of the MA experiment. The colony nucleus was maintained by Janvier Laboratories, who supplied the founder pair. From these mice, we carried out an expansion phase to establish the MA lines. In parallel, embryos descended from the founder pair were frozen and revived at generation 16 and bred as control lines after an expansion phase.

Mice can be bred with high numbers relatively quickly and can act as model mammalian species to address this question. The mutation rate per nucleotide site per generation in mice is around 50% of the value observed in humans [34–36], implying that 20 generations in the mouse equates to around 10 generations or about 250 years in humans. In this study, we carried out the first MA experiment in a mammal with a genetically characterised inbred mouse strain that has replication in the form of multiple MA lines maintained by full-sib mating. Our experiment also includes a contemporary comparison of the MA lines with a cryopreserved control that had undergone minimal mutational accumulation. This is an important feature that allows us to attempt to distinguish genetic from environmental change. We studied the impact of spontaneous mutation accumulation on the mean and genetic variation for several quantitative and life history traits using an MA experiment consisting of 55 lines maintained for 21 generations by brother-sister mating (Fig 1). The founders for the MA experiment were a single pair from one litter of the nucleus colony of a commercial laboratory whose ancestors had been maintained for several hundred generations by brother-sister mating. By Illumina sequencing, we previously showed that the nucleotide variation in the ancestral pair was close to its expectation under mutation-drift balance [37].

## Results

### Mutation accumulation in inbred mice

**Fig 2:**
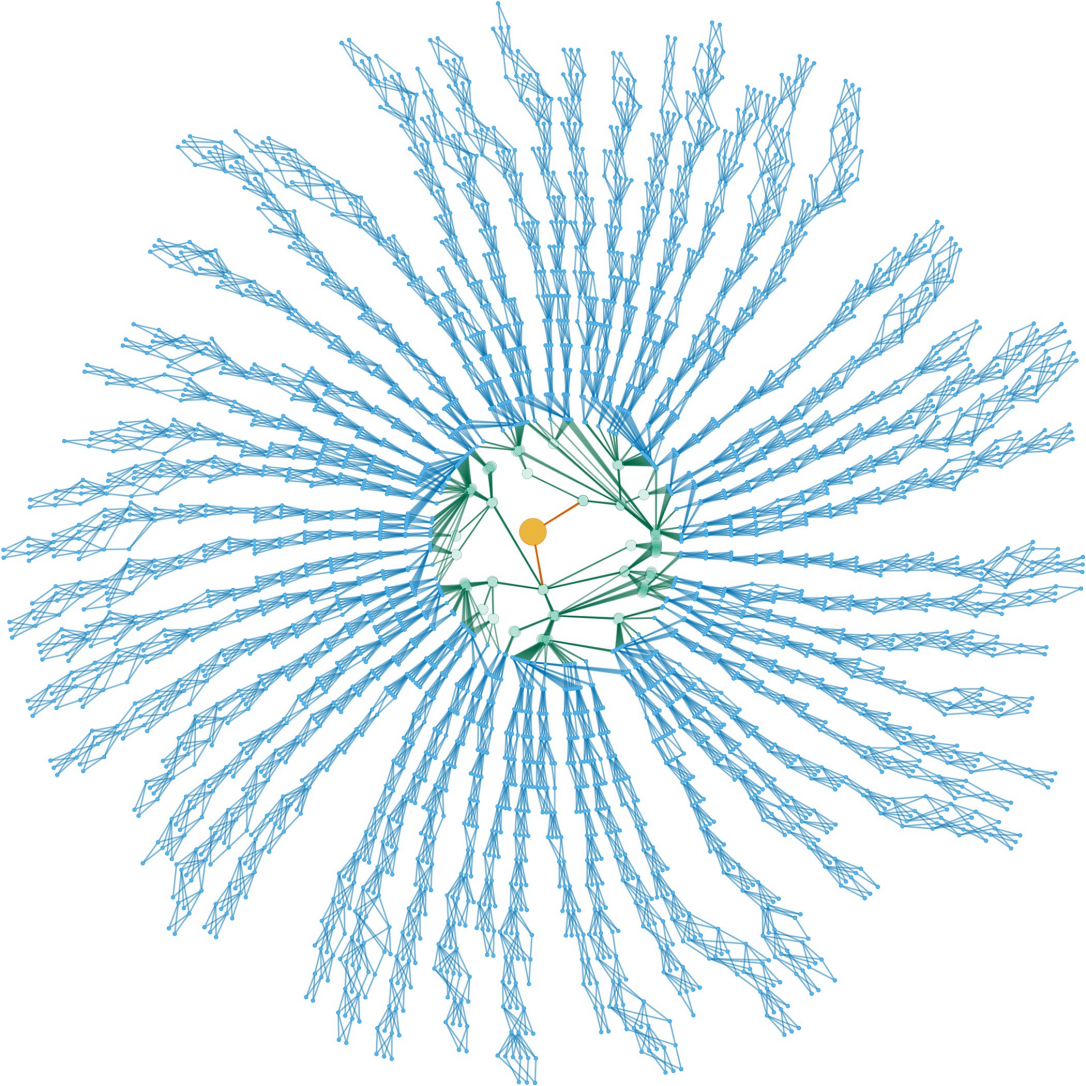
The pedigree of the MA lines. The founder pair is shown in yellow at the centre. Their immediate descendants and the expansion phase of the experiment are shown in green. The established separate MA lines are shown in blue, represented as the females that had offspring.

To study the impact of mutation accumulation on quantitative traits in mice, we initiated 55 inbred lines of the C3H/HeNRj strain and maintained them for a total of 21 generations. Our experimental design aimed to minimise, as far as possible, the influence of natural selection. Starting with the founder pair (designated generation zero), mice were bred by brother-sister mating to expand the cohort over 3 generations in order to establish the MA lines (Figs 1, 2 and Fig A in S1 Text). The founders were a brother-sister pair of an inbred strain that had previously been maintained by full-sib mating for several hundred generations [38]. The mice have therefore been in the laboratory environment for many decades and became adapted to it during and after the process of inbreeding. The supplier (Janvier Laboratories, https://janvier-labs.com) provided the 2 founder mice from their nucleus colony, which are also maintained by brother-sister mating. Note that in the case of mice derived from a commercial production colony, brother-sister mating is not guaranteed in their immediate ancestors [37]. In order to determine whether the amount of nucleotide variation within and between the 2 founders matched the expectation of a line maintained by full-sib mating, we sequenced the 2 mice by Illumina technology. A total of 130 SNPs were detected, and this level of variation implies a mutation rate of 7.9 × 10^−9^ [37], which is consistent with other direct estimates [34,35,39,40]. Details of the SNPs detected are provided in [37].

Three pairs of mice were mated per MA line each generation. There were a total of 55 MA lines established, which by generation 18 had declined to 51 MA lines. To breed the next generation, pairs of mice were picked at random from a single randomly picked litter. If insufficient pairs could be picked from the first litter, substitutes were randomly picked from a second or third randomly picked litter.

Embryos from descendants of the founder pair were stored in a cryopreserved state for use as control lines (Fig 1). To produce the control lines, the stored embryos were thawed, and grown up in recipient mothers when the MA lines were at generation 16 of the MA experiment. Control line mice were exposed to the bedding of the MA lines for one generation in order to expose the mice to the microbiota of the MA lines. The number of control individuals was expanded over 2 generations, and 20 control lines were maintained for a further 3 generations under the same breeding scheme as the MA lines. Phenotypic measures for the control lines were taken contemporaneously with the MA lines (see Methods for details).

### Genome sequencing of the MA lines

A mouse from each MA line was sequenced by Illumina technology at generation 8 or 9. Based on the number of de novo variants detected (i.e., variants observed unique to a MA line and not present in the founder pair), and the application of a mutation dropping algorithm to account for the effects of sampling of mutations in a known pedigree, the single-nucleotide mutation rate was estimated to be 6.3 × 10^−9^ per nucleotide site per generation [74]. This is typical of rates estimated for the house mouse [37,40] and approximately 50% of the average value for humans [36].

### Changes of mean values for quantitative traits

**Fig 3:**
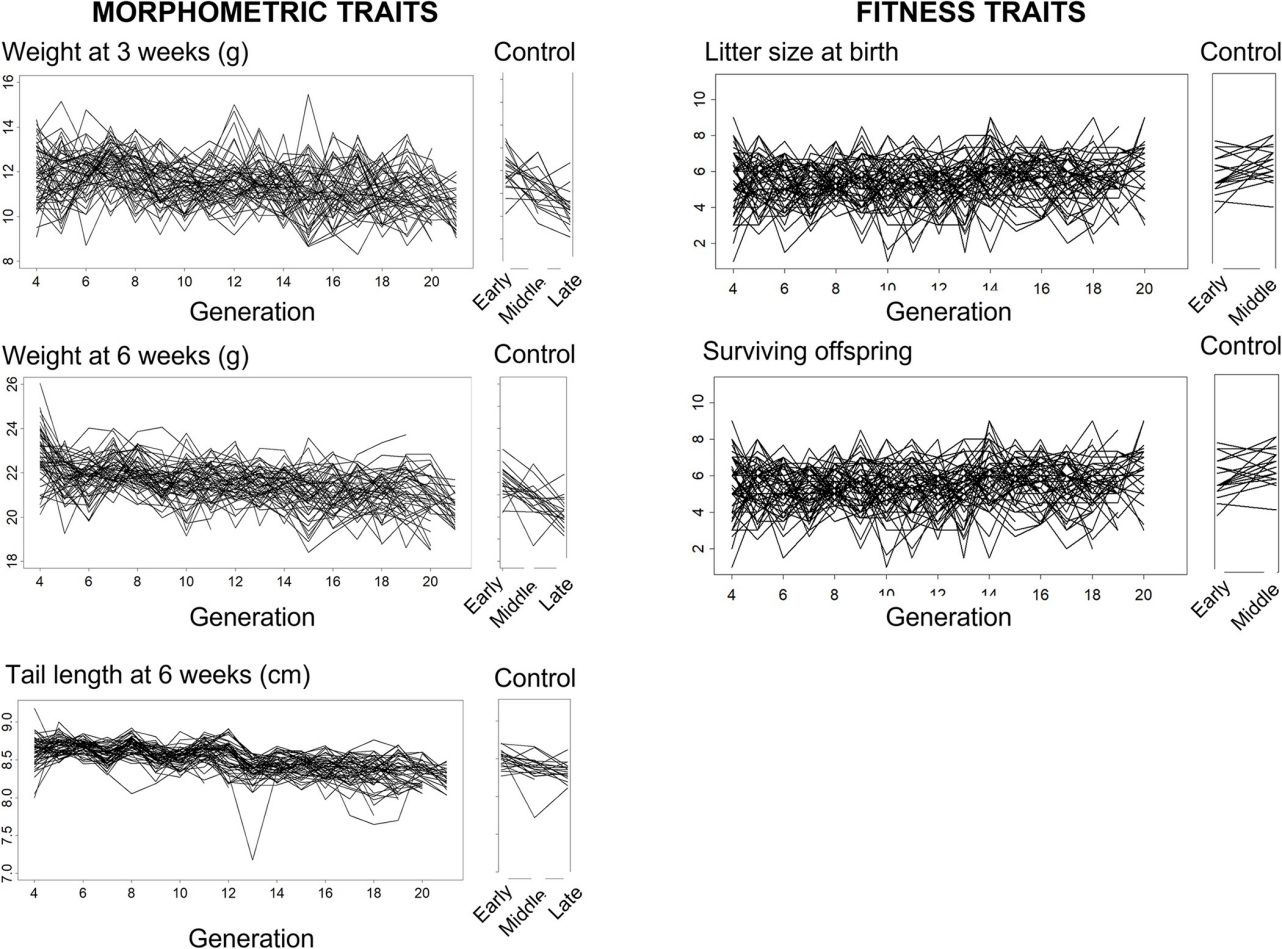
Average trait values of each MA and control line plotted against generation number (the first 3 and 2 generations of the MA and control experiments, respectively, were used to breed separate lines from founder individuals in an expansion phase, and therefore are not included). The values for the morphological traits are averages for the 2 sexes. For the fitness-related traits, litter size at birth and surviving offspring, values belong only to mothers, and therefore there are no data for these traits in the final generation. The data underlying this figure can be found in https://zenodo.org/records/12783268.

**Fig 4:**
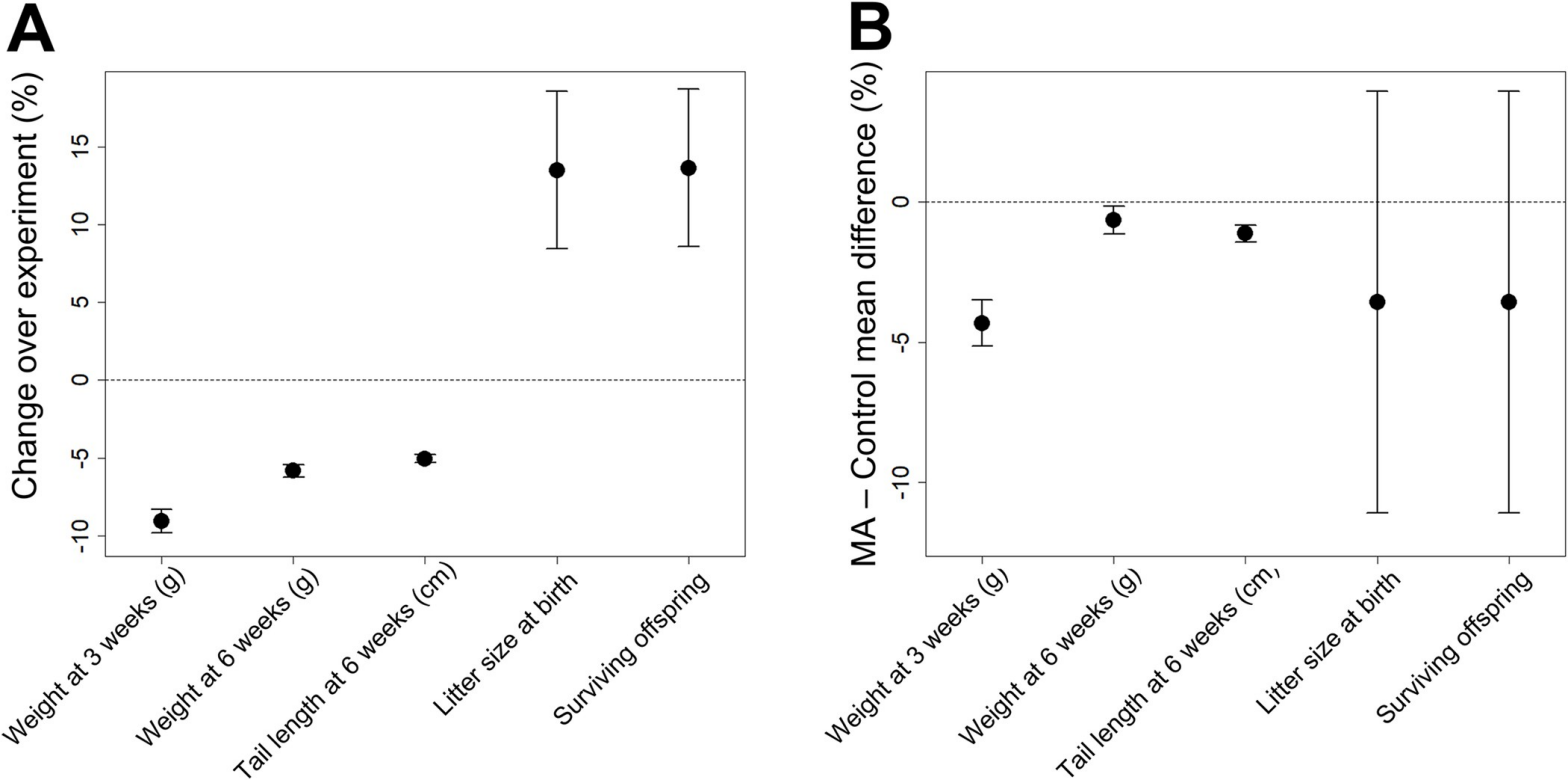
**(**A**)** The percent change in trait value over the experiment and its 95% confidence interval for the 3 morphometric traits (body weight at 3 and 6 weeks and tail length at 6 weeks) and the two fitness-related traits (litter size and number of surviving offspring) estimated using linear models, with generation as a fixed effect and sex and litter size fitted as additional fixed effects in models with morphometric traits. **(**B**)** Differences between phenotypic means of MA lines and controls for overlapping time periods (mice born and bred during the same time period), expressed as percent difference with 95% confidence intervals. The data underlying this figure can be found in https://zenodo.org/records/12783268.

After establishment of the MA lines, for every mouse in the pedigree we measured the morphological traits body weight at 3 and 6 weeks of age and tail length at 6 weeks of age, and for each mating we recorded the life history traits (traits related to fitness) litter size at birth and number of offspring surviving to 3 weeks of age. The mean values plotted for each MA line (Fig 3) and linear regression of trait values on generation number suggest that litter size and number of surviving offspring increased over the 21 generations of mutation accumulation, whereas there were downward trends for the 3 morphological traits (Fig 4A and Table A in S1 Text).

Changes in the phenotypic means over time could be due to genetic or environmental changes (including epigenetic changes) or both. Similarly, an absence of phenotypic change could be a consequence of genetic and environment change cancelling each other out. To distinguish between these possibilities, we compared the phenotypic means of the MA lines with the means of the control MA lines for individuals born within the same time period while controlling for sex and litter size in the case of the morphological traits (Fig 4B).

For all traits, MA lines had lower means than control lines, especially weight at 3 weeks. For the morphological traits, the 95% confidence limits for differences between the means did not overlap zero (Fig 4B and Table B in S1 Text).

For the 3 morphological traits, however, the differences are substantially smaller than the changes in phenotypic means observed over the 21 generations of mutation accumulation (Fig 4A and Table A in S1 Text). For example, tail length decreased in mean by 5.0% over the course of the MA experiment (Fig 4A), but the difference between the controls and the MA lines was only 1.1% of the control mean (Fig 4B). This suggests that for the morphological traits, the changes in phenotypic means that occurred as the experiment progressed were largely environmental rather than mutational in origin. For litter size and number of surviving offspring, however, the differences between the control and the MA lines are negative (Fig 4B) and in the opposite direction to the slope of the linear regression of MA line means on generation number (Fig 4A and Table A in S1 Text). For example, litter size at birth increased in mean by 13.5% over the course of the MA experiment, but the difference between the controls and the MA lines was only 3.6% of the control mean. This suggests that environmental changes increasing litter size and survival over the course of the MA experiment more than cancelled any decreases in phenotypic means attributable to mutation accumulation. Note that almost all of the impact of mutation accumulation was on litter size, and survivorship after birth to 3 weeks of age in both the controls and MA lines was close to 100%.

### Changes of variance and evolvability

Variation from new mutations also has the potential to sustain response to artificial selection in farm animals and crops [16]. The response from mutations is expected to be proportional to the product of the new heritability arising each generation, *h*^2^_*M*_, and the effective population size. Previous estimates of divergence between inbred lines of house mice or selection response in inbred lines suggest *h*^2^_*M*_ values as high as 1% per generation [14,15]. We estimated *h*^2^_M_ for the different traits by two methods. Firstly, using the “Animal Model” incorporating the mutational covariance matrix, which uses information in covariances between every pair of individuals in the pedigree, and secondly, by a “mutation dropping” approach (see Methods) that only uses information in between-MA line variation (Table 1).

**Table 1:** Mutational heritability estimates with their 95% confidence intervals (CIs) from 2 methods, along with the mean-standardised mutational coefficient of variation, *CV*_*M*_, a rescaled *CV*_*M*_ intended to account for positive correlations among dimensions in multidimensional traits, and another measure of evolvability, *I*_M_. These last 3 metrics were calculated using the Animal Model estimates of mutational variance (see Methods for more details).

	*h*^2^_M_ × 10^−3^		*CV*_M_ × 10^−3^	Rescaled *CV*_M_ × 10^−3^	*I*_M_ × 10^−3^
Trait	Animal model [95% CIs]	Mutation drop [95% CIs]	Animal model [95% CIs]		
Weight at 3 weeks	1.7 [~0.0, 3.5]	2.5 [1.5, 6.6]	5.34 [0.02, 7.87]	1.780	0.0285
Weight at 6 weeks	9.3 [0.6, 15.0]	3.1 [1.1, 8.1]	6.10 [4.08, 7.63]	2.032	0.0372
Tail length at 6 weeks	2.2 [1.2, 3.7]	4.4 [2.7, 16]	1.94 [1.39, 2.24]	0.972	0.00378
Litter size at birth	1.2 [0.49, 2.8]	−0.0 [−4.0, 9.2]	11.97 [7.72, 18.03]	11.97	0.143
Surviving offspring	1.2 [0.59, 2.8]	0.2 [−3.9, 8.5]	11.78 [8.49, 18.23]	11.78	0.139

The mutation dropping approach does not depend on covariances between close relatives and therefore should not be subject to bias caused by the presence of environmental covariance between closely related individuals, which might be unaccounted for in the Animal Model approach. However, estimates are more imprecise because less information is used. The mutational heritability for weight at 6 weeks is nearly 1% and is higher than the mutational heritability estimated at 3 weeks of age, presumably because environmental variance associated with maternal effects is higher at 3 weeks (Table C in S1 Text). The mutational heritability for weight at 6 weeks is therefore of the same order as previous estimates [14,15], implying a ~1% increase in heritability per generation (for the Animal Model). The mutational heritability for tail length is lower, perhaps reflecting a smaller mutational target. Life history traits are presumably subject to higher environmental variance and estimates of *h*^2^_M_ have higher uncertainty. However, the mutational coefficient of variation for the life history traits are higher than for the morphological traits, consistent with other studies [12]. All *h*^2^_M_ estimates are consistent with what is observed in a range of species [13]. The removal of the numerator relationship matrix from the mixed model analysis for estimating the additive genetic variance in the base population had almost no effect on the estimates of *h*^2^_M_ (Table D in S1 Text). This is as expected for strains of mice that have been inbred for hundreds of generations.

Two metrics of evolvability, the rescaled mean-standardised mutational coefficient of variation, *CV*_*M*_, and the expected proportional change under one unit strength of selection, *I*_M_, are calculated for each trait for more meaningful comparisons among traits and with other studies (Table 1), although caution should be taken to ensure measures of evolvability in other studies are calculated appropriately [41]. After rescaling, the mutational coefficient of variation remained higher for life history than morphometric traits. Lastly, a comparison of the *I*_M_ measure of evolvability implies that the same strength of selection on life history traits would result in an expected change that is 4 to 5 times larger than that of the change in weights at weeks 3 and 6, and almost 40 times larger than the change expected in tail length. The difference between life history and morphological traits (and between weights and tail length) in their evolvabilities is likely due to the latter’s smaller mutational target size, which may be connected with differences in mutational effect distributions rather than numbers of loci underlying the traits [13,42].

## Discussion

We carried out the first MA experiment in a mammalian species with multiple inbred lines and a cryopreserved control. In spite of having a substantial amount of replication and the long duration of the experiment (approximately 7 years), estimates of the mutational variation have wide confidence intervals. However, point estimates suggest that the new heritability arising from mutation for growth rate in mice is substantial and would imply that mutation makes a substantial contribution to the selection response for analogous traits in farm animals [16,17]. The time scale over which new mutations contribute to selection response depends critically on the nature of the distribution of effects of mutations [43], and our experiment provides no direct information on this. Under an additive model, the contribution to the selection response from mutations is proportional to the effective population size, implying that large populations are better able to utilise this form of variation [16]. Such populations are also less likely to suffer the potentially deleterious pleiotropic effects of recessive mutations.

Our MA experiment made use of a cryopreserved control, which we assume had undergone minimal mutation accumulation. We observed 3.6% decreases in litter size and number of surviving offspring compared to the control (with a lower confidence limit implying a reduction of 11%). Assuming that these changes occurred over 16 generations (the MA line generation when the control lines were initiated), this implies decreases in the trait means of 0.22% per generation (with a lower confidence limit implying a reduction of approximately 0.7%). What can we infer from these changes about the potential decay of fitness traits in humans from spontaneous mutation accumulation? A difficulty of making such an inference is that traits analogous to litter size and number of surviving offspring do not exist in humans. We therefore use the number of surviving offspring as a trait related to total fitness. One way of predicting the maximum plausible change in fitness due to mutation accumulation in humans is to multiply the observed change in the number of surviving offspring in the mouse by the ratio of the human to mouse per nucleotide site mutation rate. This implicitly assumes that the net fitness effects of mutations in humans and mice are of a similar magnitude. The ratio of the single-nucleotide mutation rates is somewhat less than a factor of 2 [6,37,40]. We therefore predict a reduction in mean fitness from mutation accumulation of about 0.38% per generation in humans. Over a period of 200 years, or about 8 generations, the predicted reduction in fitness in humans would therefore be approximately 3%. However, for a number of reasons the impact of mutations in humans is likely to be substantially smaller than this value implies. First, our MA experiment involved deliberate inbreeding by brother-sister mating, which leads to the fixation of deleterious mutations, whereas in an outbred population (like human populations) recently arisen deleterious mutations are almost exclusively in the heterozygous state. Theory and empirical evidence suggest that deleterious mutations are partially recessive, on average [44,45], and the heterozygous effects of mutations determine the short-term loss of fitness under mutation accumulation. Net recessivity reduces the effect on fitness of mutation accumulation in an outbred population compared to that expected in an inbred line by as much as 50% [45]. Second, although it is highly likely that the strength of natural selection has lessened in many modern human populations, natural selection continues to operate in all human populations. For example, sexual selection has presumably not lessened, and there is considerable variation in family size on which selection can continue to operate. This is corroborated by evidence of directional and stabilising natural selection operating on various quantitative traits in current human populations [46]. Third, the distribution of fitness effects of mutations has been inferred to be highly leptokurtic [47], and much new mutational variation is explained by large-effect mutations. These mutations will tend to be quickly eliminated by natural selection (although they might reach higher frequencies if selection is reduced), and empirical evidence suggests that their impact is strongly attenuated even in populations of effective size of 10 and greater [21]. Finally, the C3H mouse strain studied in the present experiment has active intracisternal A-type particle (IAP) retroelements. Although not classified as a mutator strain, the number of insertions per generation likely exceeds the number of transposable element insertions in other strains of mice and in humans [48,49]. Their presence therefore potentially generates genetic variation for quantitative and life history traits [50].

Our experimental design did not assess the fitness effects of mutation accumulation in a natural environment, but the mouse strains used are expected to be adapted to the benign laboratory breeding environment. While some studies suggest that fitness effects of mutations are accentuated in harsh environments [51], the overall picture is less clear. Some studies imply a larger and some studies imply a smaller effect on fitness of mutation accumulation in stressful environments [52]. This, however, is an important caveat. We do not know if the fitness effects of mutations would be substantially higher if the mice had to express their full repertoire of behaviour and physiology.

In summary, although we observed reductions in the means for fitness traits in MA lines of mice relative to control line mice, the magnitude of the reduction in fitness suggests that it is doubtful whether a reduction in mean fitness due to mutation accumulation will be observable in human populations in the near future. Undoubtedly, of far greater concern is the unsustainable overexploitation of finite global resources against a background of an expanding human population.

## Materials and methods

### Ethics statement

Maintenance and handling of the animals were conducted in accordance with German Animal Welfare Act and FELASA guidelines. The project was approved with the number 1158 by the Animal Welfare Officers of the University of Kiel according to the German Animal Welfare Act §4 “Killing animals and organ withdrawal for scientific purpose.” Permits for keeping mice were obtained from the veterinary office “Veterinäramt Kreis Plön” under permit number: PLÖ-000 4697 (08.04.2014).

### Mouse breeding and trait measurement

One pair of full sibs of the C3H/HeNRj strain was obtained from embryos directly from Janvier Labs’ colony nucleus. This pair of mice (henceforth referred to as the “founders”) are the descendants of hundreds of generations of brother-sister mating [38] and are therefore expected to be nearly isogenic. This was verified by Illumina sequencing, showing an amount of variation in the founders consistent with mutation-drift balance [37]. The founders were bred together and their offspring used in an expansion phase of successive full-sib matings for 3 generations to produce 55 separate inbred lines. To produce the next generation, one of these 3 matings was selected at random and males and females from this mating were selected at random to set up, up to 3 brothers-sister matings. If less than 3 matings could be set up using the first family, then randomly selected individuals from the second or third families were used. If it was not possible to produce 3 brother-sister matings then males and females from different families (but within the same line) were mated. The frequency of such matings using males and females from different families of the same line was 13%. The inbred lines were maintained for 21 generations in total.

Individual mice were weighed at 3 and 6 weeks of age, and the length of their tails measured at 6 weeks. The number of pups born was used as a measure of litter size and the number of pups born that survived to weaning age (3 weeks) was also recorded.

### Rearing environment

Mice were housed in Green Line GM500 IVC cages from Tecniplast (Italy) at the Max Planck Institute for Evolutionary Biology in Plön, Germany. The cages contained food (1328 forti, Altromin, Germany), water, bedding (aspen, Rettenmaier, Germany), nesting material, and shelter. All materials were sterilised before contact with the mice. Environmental conditions included a room temperature of 22°C +/− 2°C, humidity of 55% to 60% and room ventilation of 16 turnovers/hour. The mice were always handled under an air ventilated clean bench. The cage change was weekly. The mice facility is regularly germ tested by sentinels.

### Control lines

The founding pair was mated on March 1, 2016 and their descendants bred by brother-sister mating for 3 generations at which point embryos were obtained from superovulated females and cryopreserved and stored at Janvier Laboratories.

In September 2021, embryos were implanted into B6CBA/F1 pseudo-pregnant females and a total of 12 males and 6 females were imported into the animal facility at the Max Planck Institute. These individuals were bred together to produce 20 separate control lines in the same manner as described above, except that inbred lines were only maintained for 5 generations in total to limit mutation accumulation. The same traits were measured as described for the MA lines.

The pedigrees were checked for completeness and processed into a graph using *purgeR* v1.8 [53] and visualised with a circular tree layout with *igraph* v1.5.1 [54], using the R language v4.2.2 [55].

### Changes in trait means

Changes in trait values over generations for each trait were analysed using linear models in RStudio [56] with trait values as the response variables and generation as a fixed effect. In models with morphometric traits (weight and length) as response variables, sex and litter size were included as additional fixed effects (fitness traits, like litter size at birth and surviving offspring, are assigned only to mothers). The resulting estimates for generation (estimates of the slope of the trait values over generations) were divided by their respective mean trait values across the whole experiment and multiplied by 100 in order to obtain the percent change of trait value per generation. In order to estimate 95% confidence intervals (CIs), standard errors (SEs) of the estimates were divided by their respective mean trait values and multiplied by 100 before being doubled and added or subtracted from their respective per generation percent change in trait value.

There are methods for combining the changes of mean and genetic variance (see below) to estimate the genomic mutation rate and the distribution of effects of mutations [18,19]. However, parameter estimates are confounded with one another and statistical power tends to be low [57].

### Trait mean comparisons with control lines

**Fig 5:**
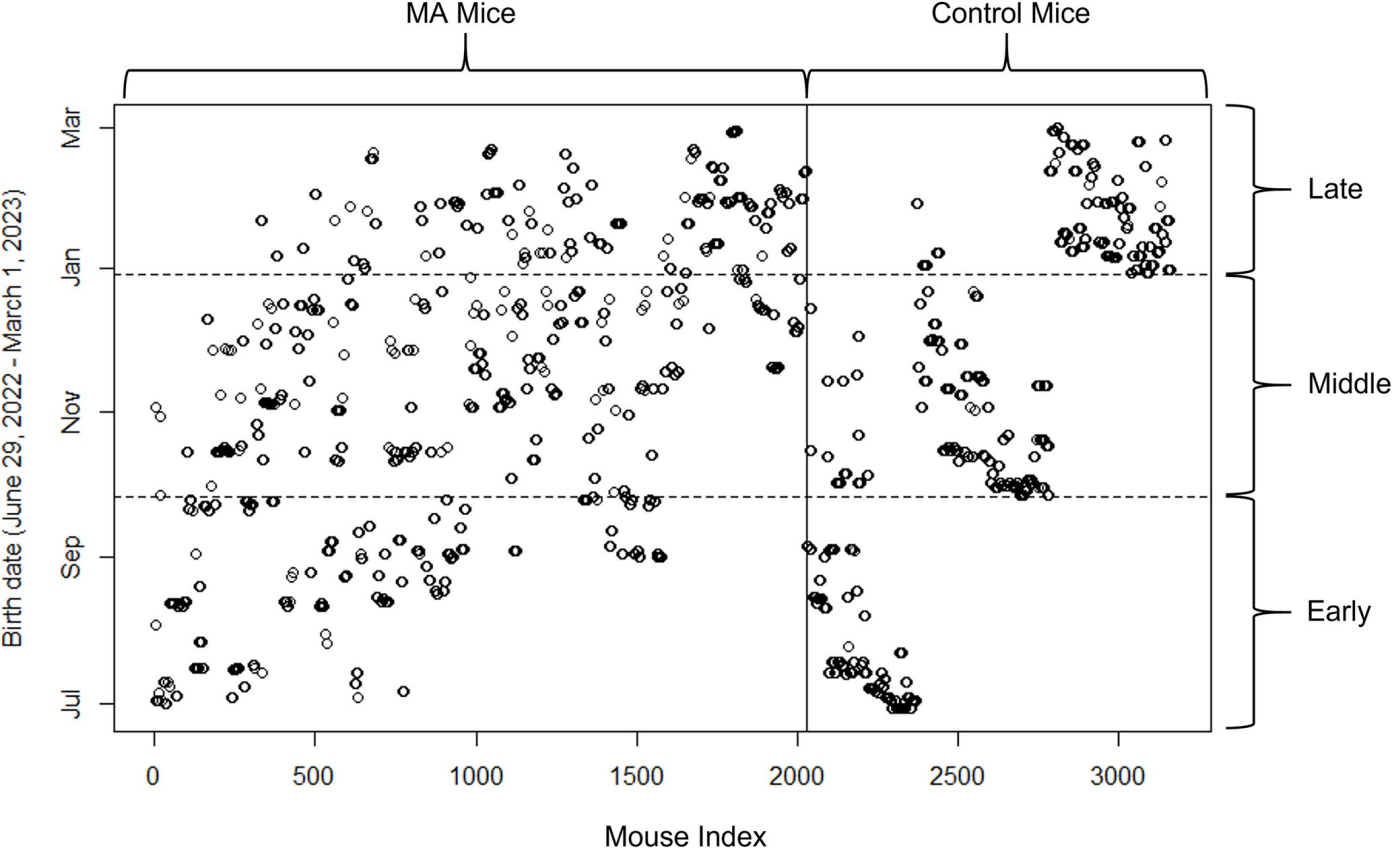
Birth dates of mice used in contemporary phenotypic comparison of MA (2,030 mice) and Control (1,132 mice) lines. The vertical (solid) line separates MA line mice (left) from Control line mice (right). The horizontal (dashed) lines separate the 3 time periods used in the analysis. Note that the control mice underwent an expansion phase and this will mitigate the influence maternal effects. The data underlying this figure can be found in https://zenodo.org/records/12783268.

To compare MA line mice with control line mice in similar environments, phenotypic values were only compared with the MA line mice that were born in the time period beginning with the birth of the first control mice after the expansion phase (generation 3 of the control lines; June 29, 2022) and ending with the birth of the last mice in generation 5 of the control lines (March 1, 2023). The MA line mice data were split into 3 time periods (early, middle, and late) which correspond roughly to the 3 generations of control line mice (Fig 5). The early time period included data from mice born between June 29, 2022 and September 26, 2022. The middle time period included data from mice born between September 27, 2022 and December 29, 2022. The late time period included data from mice born between December 30, 2022 and March 1, 2023. To determine whether there were differences between main MA line and control line means, linear models were run in R [55], as was done when examining the changes in trait means over generations (see above), except that time period was used instead of generation, and experiment was included as an additional fixed effect, which distinguished whether an individual value was from the MA line experiment or the control lines. Percent differences between MA lines and control lines were calculated by first subtracting the estimated effect of MA line experiment from the control line means for each trait, then dividing by their respective control line trait means, and multiplying by 100. Their CIs were calculated in the same way as the changes in trait means described above.

### Genetic variance in founders when analysing pedigree data

For inference of mutational heritability by the Animal Model and by gene dropping (next two sections), an assumption requires to be made about the amount of genetic variation in the founder pair. For the two analysis approaches described below, we incorporated 20 generations of full-sib mating with no trait data leading up to the founder pair. This implies that the genetic variation in the founders would be at mutation-drift balance.

### Mixed model analysis using the Animal Model modified for mutational heritability

For each trait, we estimated components of variance using a univariate animal model,

$$y=X\beta +Z_{1}u_{Litter}+Z_{2}u_{Maternal}+Z_{a}a+Z_{m}m+e,$$ (1)

where **y** was the vector of trait values, **e** was the vector of residuals, while accounting for fixed (**β**), environmental (**u**_**Litter**_ and **u**_**Maternal**_), and additive genetic effects (**a** and **m**). **β** accounted for the fixed effects: sex, generation, and litter size. Two environmental sources of variation common to all pups were fitted to model; the environmental effect of the litter (**u**_**Litter**_) and the non-genetic effect of the mother (**u**_**Maternal**_). **X** was the design matrix that related the phenotypic data to the fixed effects; sex, generation, and litter size. **Z**_**1**_, **Z**_**2**_, **Z**_**a**_ and **Z**_**m**_ were the design matrices that relate the phenotypic records to the random effects of litter (i.e., the common rearing environment), mother, additive genetic and de novo additive genetic (mutation), respectively. The additive genetic effect, **u**, was partitioned into **u** = **a** + **m**, the breeding value inherited from the genetic variation in the base generation, **a**, and from the additional genetic variation from new mutation, **m** [58,59]. The variance components for additive and mutation effects were calculated as the variance in breeding values scaled by the corresponding relationship matrix *V*_*A*,*0*_ = **A***σ*^*2*^_*a*_ and *V*_*M*_ = **M***σ*^*2*^_*m*_ [60,61]. The relationship matrix, **A**, for scaling additive genetic variance in the base population (i.e., at generation 0), *V*_*A*,*0*_, is *n* by *n* matrix, where *n* is the number of individuals in the pedigree and each element of the matrix holds a value that is the average genetic relatedness between a pair of individuals. The mutational relationship matrix, **M**, for scaling additive genetic variance accumulated over the generations in the pedigree (i.e., from generation 0 to the second last generation, *t*), *V*_*M*_, is constructed by the addition of successive **A** matrices, as [59]:

$$M=\sum_{k=0}^{t}A_{k}.$$ (2)

The model in Eq (1) was implemented in *ASReml-R* v4.1 [62] and inverse numerator relationship and mutational numerator relationship matrices (**A**^**-1**^ and **M**^**-1**^) were produced using the *nadiv* package v 2.17.2 [63] in *R* v 4.2.2 [55](see Methods in S1 Text for example script). Additional models were implemented that only differed from model (1) (i.e., the full model) in that one of the random effects were removed in order to perform a model comparison using log likelihoods, but model (1) performed the best for all traits (i.e., had the highest log likelihood) so only the variance estimates for the model (1) are discussed (Table D in S1 Text).

Mutational heritability estimates, *h*^2^_M_, were then calculated by dividing the *V*_*M*_ estimates by the total variance. The mean-standardised mutational coefficient of variation, *CV*_*M*_, was calculated by taking the square root of *V*_*M*_ and dividing it by the mean trait value, and 95% CIs for *h*^2^_M_ and *CV*_*M*_ were found by bootstrapping 1,000 times over MA lines, each time re-running the model, and taking the highest and lowest 2.5% estimates over all runs. In order to make better comparisons among traits of different dimensions (i.e., to deal with positive correlations among dimensions, which inflate CVs), rescaled *CV*_*M*_ values are provided where volumes (weights) were divided by 3 and tail length was divided by 2 [64]. The mutational evolvability, *I*_*M*_, which can be thought of as the expected amount of change in a trait due to new mutations in response to one unit of selection, was calculated by dividing *V*_*M*_ by the square of the mean trait value [11,65].

### Inference of mutational variation using between line variation by mutation dropping into the pedigree

We developed an approach to estimate the mutational heritability using the variation among line means (available at https://sourceforge.net/projects/drop-mutations-into-pedigree/). Unlike the Animal Model method, the approach does not use information in phenotypic covariances between closely related individuals. The between line divergence is simulated under a highly polygenic model, assuming normally environmentally distributed environmental effects, and the simulated between line variances each generation are matched to the corresponding observed between line variances each generation.

We first calculated a vector **z** of observed between MA line phenotypic variances each generation calculated from the phenotypic means by generation for each MA line, with a correction for the effect of sex applied. The dimensions of **z** were *t*_*2*_—*t*_*1*_ + 1, where *t*_*2*_ is the last generation of the MA experiment, and *t*_*1*_ is the first generation where data are available for the independent MA lines in the experiment.

Then, using the complete pedigree from the MA experiment, including 20 generations of full-sib matings leading up to the ancestral pair of the experiment, we simulated the fates of 10^6^ mutations dropped into this pedigree assuming diploid autosomal mendelian inheritance. We assigned each mutation a value of -*a* or +*a* with equal probability, such that the value of *a* arbitrarily produced value of 1 for the mutational variance, *V*_*M*_, as follows. For the *n* individuals in the pedigree, each individual received an average of *b* = 10^6^/*n* mutations, which could then be inherited by the individual’s descendants. Since *V*_*M*_ = *ba*^2^/2 = 1, the value of *a* = ✓(2/*b*).

Assuming an additive model, we then calculated the genotypic value of each individual in the pedigree by summing the effects of the mutations it carried. From these individual genotypic values, we then calculated an expected between MA line genetic variance vector **g** which has the same dimensions as the observed between MA line phenotypic vector **z**. We then computed an expected environmental variance vector **e** of the same dimensions as **z** by assigning to each individual an environmental effect *e* assuming *V*_*E*_ = 1. We calculated the mean environmental effect for each MA line each generation, and, based on the mean of 100 replicates, computed the expected between MA line environmental variance vector, **e**.

To estimate the mutational heritability, we minimised the sum of squares between *x***g** + *y***e** and the observed vector **z** of between MA line phenotypic variances, where *x* and *y* are parameters to be estimated. The estimate of mutational heritability = *h*^2^_*M*_ = *x/y*.

### Detection of single-nucleotide mutations

We estimated the single-nucleotide mutation (SNM) rate per site per generation by whole-genome sequencing (WGS) of each MA line. We also sequenced the founders of the MA lines to allow the identification of mutations that arose de novo uniquely in one descendant MA sample.

DNA was extracted from liver tissue of one randomly chosen male from each of 47 MA lines (8 of the 55 samples were removed due to evidence of possible DNA contamination) either at generation 8 or 9 by a standard salt extraction method that included an initial Proteinase K digestion step.

WGS was performed using the Illumina NovaSeq Platform at Edinburgh Genomics (Edinburgh, UK). The sequencing libraries were generated using a PCR-free approach yielding ~30× coverage, on average, of 150-bp paired-end sequences. Reads were aligned to the *Mus musculus* reference genome (GRCm38.p6) using BWA mem v0.7.13-r116 [66], and alignment data for each individual were processed through the following pipeline. The reads were sorted using Samtools v1.9 [67], the read mate-pair information was synchronised using “fixMateInfo” from the Picard Tools v2.2 suite [68], read groups were replaced using “setReadGroups,” and duplicate reads were marked using “markDuplicates” from Picard Tools. The processed data were indexed using Samtools.

After the alignment processing, variants were called for individual samples using HaplotypeCaller from GATK v4.1.2.0 [69], with options to enable calling at variant and invariant sites (using the option “—emit-ref-confidence BP_RESOLUTION”). Variant calls were then combined into one variant call format (VCF) file per strain using GATK’s CombineGVCFs. The final sets of variants were called from these VCFs, together with invariant sites, with GATK’s GenotypeGVCFs using the option “—include-non-variant-sites.”

We only considered single-nucleotide variants. Candidate de novo mutations were identified from the set of sites where variation was unique to one MA sample and where variation was absent from the founder mice. Each candidate variant was further filtered according to the following criteria:

1. Phred-scaled quality score for the variant (QUAL) ≥ 30.
2. The read depth of every sample ≥10.
3. The read depth of every sample <60.
4. If the mutation was called as heterozygous, the proportion of reads supporting it was in the range [0.25, 0.75].
5. The total number of variant reads in non-mutated MA samples did not exceed an impurity threshold of 25 reads (see below).

These criteria were coded into Cython scripts, which incorporated the Python wrapper cyvcf2 0.30.18 [70]. The variant sites that passed the above criteria were then subjected to a manual verification using the Integrative Genomics Viewer v2.16.0 (IGV [71]). Mutations were rejected if they lacked unambiguous support from the read alignments or were not unique to a single MA line. In addition to the above requirements, we filtered candidate mutations according to the following criteria.

6. Variants are not in phase with other variants.
7. Variants in sex chromosomes are homozygous.
8. The variant site has no more than 2 alleles.
9. Variants do not have more than 1 read whose read pair was aligned to another chromosome.

The threshold value for read impurity defined in criterion 5 was determined by increasing the number of impurities allowed (starting from zero) until the number of new true positive candidate mutations included no longer increased as the number of impurities allowed was increased. Haplotype phase distance between variants in criterion 6 was determined by GATK v4.1.2.0 HaplotypeCaller “active site” defining algorithms [72]. Criteria 3 and 6 through 9 were specifically intended to filter out false positives due to misaligned paralogous reads. Many regions containing misaligned paralogous reads can be recognised because they tend to contain groups of linked variants in phase.

To estimate mutation rates with single-nucleotide precision, we defined a fraction of the genome as “callable.” Here, the callable genome was determined following the filtering criteria 1 to 3 defined above, which can be applied to invariant as well as variant sites, so that the callable genome has an equivalent quality as was required to detect mutations. The callable sites were restricted to the autosomes and the X chromosome. The mouse Y chromosome consists almost entirely of ampliconic genes that are arranged in tandem units [73], and was excluded, since mutations were effectively unmappable.

The mutation rate was calculated by dividing the number of mutations by the product of twice the number of callable sites (due to diploidy), the number of generations, and the number of mice. A correction factor was applied to this mutation rate estimate to account for the potential loss of segregating variants in the mice pedigree that were not captured in our samples, and the variants that were filtered out because they were not unique to one line due to arising in the expansion phase. The correction factor was calculated using a gene-dropping simulation approach, whereby in each iteration of the simulation a mutation would appear in a randomly chosen individual in the MA pedigree, the mutation would be transmitted (or lost) by the rules of random segregation until the last generation of the pedigree, and the number of individuals heterozygous or homozygous for the mutant allele in the final generation would be counted. One million iterations of this simulation were performed to determine the expected proportion of mutations that are not captured in our MA line experiment and to calculate the correction factor to account for this.

## Supporting information

S1 Text Fig A. Partial representation of the pedigree; Table A. Trait means, and slopes of regression of MA line trait values on generation number; Table B. Control and MA line trait means; Table C. Variance component estimates from ASREML mixed model analyses; Table D. Comparison of models with log likelihoods and variance component estimates from ASREML; Supplementary Methods.
(PDF)

## Abbreviations

CI: confidence interval
IAP: intracisternal A-type particle
MA: mutation accumulation
SE: standard error
SNM: single-nucleotide mutation
VCF: variant call format
WGS: whole-genome sequencing
