# Supplementary material for: An estimate of fitness reduction from mutation accumulation in a mammal allows assessment of the consequences of relaxed selection
Source: PLoS Biol. 2024 Sep 26;22(9):e3002795. doi: 10.1371/journal.pbio.3002795 (PMC11426515; doi:10.1371/journal.pbio.3002795)
Supplement: S1 Text — Fig A. Partial representation of the pedigree; Table A. Trait means, and slopes of regression of MA line trait values on generation number; Table B. Control and MA line trait means; Table C. Variance component estimates from ASREML mixed model analyses; Table D. Comparison of models with log likelihoods and variance component estimates from ASREML; Supplementary Methods. (PDF) [file pbio.3002795.s001.pdf]

An estimate of fitness reduction from mutation accumulation in a mammal allows assessment of the consequences of relaxed selection

Jobran Chebib, Anika Jonas, Eugenio López-Cortegano, Sven Künzel, Diethard Tautz and Peter D. Keightley

Supporting Information

Fig. A. Partial representation of the pedigree of the MA experiment including the founder pair (generation 0), the expansion phase, and the 55 independent MA lines up to generation 11 (each row is a generation).

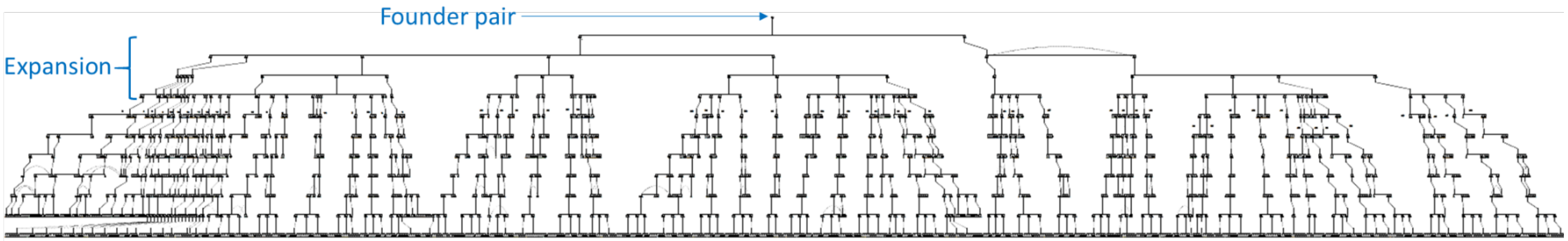

Table A. Trait means, and slopes of regression of MA line trait values on generation number, expressed in percent (for 15,598 mice over the entire experiment).

Trait	Mean	Change per generation (%)	95% CIs per gen
Weight at 3 weeks (g)	11.4	-0.535	-0.579, -0.491
Weight at 6 weeks (g)	21.4	-0.343	-0.366, -0.319
Tail length at 6 weeks (cm)	8.49	-0.297	-0.312, -0.282
Litter size at birth	5.46	0.795	0.498,1.092
Surviving offspring	5.46	0.803	0.505, 1.010

Table B. Control and MA line trait means (controlling for sex and litter size in the case of the morphological traits) for the overlapping time-period and the mean difference between them expressed as a percentage along with 95% CIs. Note: the phenotypic means for litter size at birth and surviving offspring for both control and MA line mice were almost identical during this period of overlap.

Trait	Control mean	MA mean	MA – control mean difference (%)	Lower 95% CI (%)	Upper 95% CI (%)
Weight at 3 weeks (g)	11.13	10.65	-4.32	-5.15	-3.49
Weight at 6 weeks (g)	20.83	20.69	-0.65	-1.14	-0.16
Tail length at 6 weeks (cm)	8.39	8.30	-1.13	-1.44	-0.83
Litter size at birth	6.07	5.86	-3.58	-11.1	3.92
Surviving offspring	6.07	5.86	-3.58	-11.1	3.92

Table C. Variance component estimates from ASREML mixed model analyses. $V_{A,0}$ is the additive genetic variance present in the population at generation 0, V_M is the additive genetic variance produced by new mutations during the experiment, V_{litter} is the among litter variance, $V_{maternal}$ is the variance attributed to maternal effects, $V_{residual}$ is the residual variance, h^2_A is the proportion of $V_{A,0}$ contributing to the total variance (heritability), h^2_M is the proportion of V_M contributing to the total variance (mutational heritability), and CV_M is the mean-standardised mutational coefficient of variation. h^2_A and h^2_M estimates are provided along with their respective standard errors.

Trait	$V_{A,0}$	V_M	V_{litter}	$V_{maternal}$	$V_{residual}$	V_{Total}	h^2_A (SE)	h^2_M (SE)	CV_M
Weight at 3 weeks	2.16E-06	0.00367	1.573	0.116	0.486	2.179	9.90E-07 (1.135)	0.00169 (0.00287)	0.00534
Weight at 6 weeks	3.25E-06	0.0171	0.569	0.0394	1.227	1.852	1.76E-06 (3.11E-08)	0.00923 (0.00164)	0.00610
Tail length at 6 weeks	1.90E-06	0.000273	0.0243	0.00470	0.0950	0.124	1.53E-05 (2.12E-07)	0.00220 (0.000490)	0.00194
Litter size at birth	2.95E-06	0.00428	5.68E-07	1.32E-07	3.505	3.509	8.40E-07 (2.54E-08)	0.00122 (0.000548)	0.01197
Surviving offspring	2.96E-06	0.00413	5.56E-07	9.75E-08	3.521	3.525	8.39E-07 (2.54E-08)	0.00117 (0.000536)	0.01177

Table D. Comparison of models with log likelihoods and variance component estimates from ASREML mixed model analyses. $V_{A,0}$ is the additive genetic variance present in the population at generation 0, V_M is the additive genetic variance produced by new mutations during the experiment, V_{litter} is the among litter variance, $V_{maternal}$ is the variance attributed to maternal effects, $V_{residual}$ is the residual variance, h^2_A is the proportion of $V_{A,0}$ contributing to the total variance (heritability), and h^2_M is the proportion of V_M contributing to the total variance (mutational heritability). h^2_A and h^2_M estimates are provided along with their respective standard errors. c^2_{litter} and c^2_{maternal} are the estimates of the proportion of litter and maternal variance, respectively, contributing to the overall variance. Model "Full" is model (1) described in the methods section. Other models are the same as model (1) with one of the random effects removed (e.g. "No animal" has no numerator relationship matrix, **A**, in the mixed model).

Trait	Model	Log likelihood	$V_{A,0}$	V_M	V_{litter}	$V_{maternal}$	$V_{residual}$	V_{Total}	h^2_A	h^2_M	c^2_{litter}	$c^2_{maternal}$	h^2_A SE	h^2_M SE
Weight at 3 weeks	Full	-6752.6	2.16E-06	0.00367	1.573	0.116	0.486	2.179	9.90E-07	0.00169	0.722	0.0532	1.135	0.00287
Weight at 3 weeks	No maternal	-6754.7	1.44E-05	0.00424	1.686	NA	0.484	2.171	6.65E-06	0.00195	0.775	NA	1.149E-07	0.000617
Weight at 3 weeks	No litter	-8831.0	1.06E-06	0.00255	NA	1.252	0.806	2.060	5.13E-07	0.00124	NA	0.608	1.011	0.00214
Weight	No	-	3.922	NA	1.575	0.129	0.496	6.122	0.641	NA	0.257	0.0210	0.0826	NA

at 3 weeks	mutation	6754.3												
Weight at 3 weeks	No animal	-6752.6	NA	0.00367	1.573	0.116	0.486	2.179	NA	0.00169	0.722	0.0532	NA	0.000568
Weight at 6 weeks	Full	-11161.0	3.25E-06	0.0171	0.569	0.0395	1.227	1.852	1.76E-06	0.00923	0.307	0.0213	3.11E-08	0.00164
Weight at 6 weeks	No maternal	-11161.8	3.25E-06	0.0181	0.604	NA	1.223	1.845	1.76E-06	0.00982	0.327	NA	3.12E-08	0.00168
Weight at 6 weeks	No litter	-11385.9	3.25E-06	0.0175	NA	0.426	1.365	1.808	1.80E-06	0.00966	NA	0.235	3.07E-08	0.00168
Weight at 6 weeks	No mutation	-11188.1	14.367	NA	0.578	0.0882	1.281	16.313	0.881	NA	0.0354	0.00541	0.0228	NA
Weight at 6 weeks	No animal	-11161.0	NA	0.0171	0.569	0.0394	1.227	1.852	NA	0.00923	0.307	0.0213	NA	0.00164
Tail length at 6 weeks	Full	8308.6	1.90E-06	0.000273	0.0243	0.00470	0.0950	0.1242	1.53E-05	0.00219	0.196	0.0378	2.13E-07	0.000521

Tail length at 6 weeks	No maternal	8304.9	1.90E-06	0.000298	0.0288	NA	0.0949	0.124	1.53E-05	0.00240	0.233	NA	2.13E-07	0.000522
Tail length at 6 weeks	No litter	8194.1	1.90E-06	0.00027	NA	0.0218	0.101	0.123	1.54E-05	0.00219	NA	0.177	2.08E-07	0.000488
Tail length at 6 weeks	No mutation	8294.6	0.248	NA	0.0245	0.00571	0.0959	0.374	0.663	NA	0.0654	0.0153	0.0530	NA
Tail length at 6 weeks	No animal	8308.6	NA	0.000273	0.0243	0.00469	0.0950	0.124	NA	0.0022	0.196	0.0378	NA	0.000490
Litter size at birth	Full	-2705.1	2.95E-06	0.00428	5.68E-07	1.32E-07	3.505	3.509	8.40E-07	0.00122	1.16E-07	2.15E-07	2.54E-08	0.000547
Litter size at birth	No maternal	-2705.1	2.95E-06	0.00428	5.68E-07	NA	3.505	3.509	8.40E-07	0.00122	1.16E-07	NA	2.54E-08	0.000548
Litter size at birth	No litter	-2711.5	2.95E-06	0.00620	NA	7.90E-08	3.501	3.507	8.41E-07	0.00177	NA	2.25E-08	2.57E-08	0.000671

Litter size at birth	No mutation	-2706.4	3.96863	NA	5.71E-07	4.29E-08	3.533	7.502	0.529	NA	7.62E-08	5.72E-09	0.131	NA
Litter size at birth	No animal	-2705.1	NA	0.00428	5.68E-07	8.12E-08	3.505	3.509	NA	0.00122	1.16E-07	2.31E-08	NA	0.000548
Surviving offspring	Full	-2709.6	2.96E-06	0.00413	5.56E-07	9.75E-08	3.521	3.525	8.39E-07	0.00117	1.58E-07	2.77E-08	2.54E-08	0.000536
Surviving offspring	No maternal	-2709.6	2.96E-06	0.00413	5.56E-07	NA	3.521	3.525	8.39E-07	0.00117	1.58E-07	NA	2.54E-08	0.000536
Surviving offspring	No litter	-2715.8	2.96E-06	0.00602	NA	7.40E-08	3.517	3.522	8.40E-07	0.00171	NA	2.10E-08	2.56E-08	0.000657
Surviving offspring	No mutation	-2710.8	3.778	NA	5.61E-07	5.85E-07	3.549	7.327	0.516	NA	7.65E-08	7.98E-08	0.134	NA
Surviving offspring	No animal	-2709.6	NA	0.00413	5.56E-07	7.65E-08	3.521	3.525	NA	0.00117	1.58E-07	2.17E-08	NA	0.000536

g														

Supplementary Methods

Excerpt of R code for mixed model analysis on tail length using ASREML-R

```
# CSV data file is read in as a dataframe called C3H_data
C3H_data = read.csv("C3H_MA_data.csv", header=T, sep=',')

# A pedigree is made from the animal, sire and dam columns of C3H_data
# and inverse A and M matrices are created using nadiv package
C3H_ped = C3H_data[,1:3]
ainv <- ainverse(C3H_ped)
listMinv <- makeMinv(C3H_ped)$listMinv
attr(listMinv, "INVERSE")  <- TRUE

# Variable types are explicitly assigned a type
C3H_data$animal <-as.factor(C3H_data$animal)
C3H_data$dam <-as.factor(C3H_data$dam)
C3H_data$sire <-as.factor(C3H_data$sire)
C3H_data$Gen <-as.factor(C3H_data$Gen)
C3H_data$sex <-as.factor(C3H_data$sex)
C3H_data$Litsize <-as.factor(C3H_data$Litsize)
C3H_data$tail <-as.numeric(C3H_data$tail)
C3H_data$Litter <-as.factor(C3H_data$Litter)
```

Mixed model run with all fixed and random effects included

```
asreml(fixed = tail ~ 1 + sex + Gen + Litsize, random =~vm(animal, ainv) + vm(animal, listMinv) + Litter + dam,
residual=~idv(units), data=C3H_data, na.action = na.method(x="omit", y="omit"),workspace="3gb")
```
